# Relationship Between the Vascular Bundle Structure of Panicle Branches and the Filling of Inferior Spikelets in Large-Panicle Japonica Rice

**DOI:** 10.3389/fpls.2021.774565

**Published:** 2021-12-15

**Authors:** Cuicui You, Hui Wang, Yaru Huang, Peng Xu, Liquan Wu, Fuhan Yu, Xinyue Zhong, Jin Gao, Liangliang Zhang, Haibing He, Jian Ke

**Affiliations:** ^1^College of Agronomy, Anhui Agricultural University, Hefei, China; ^2^Jiangsu Collaborative Innovation Center for Modern Crop Production, Nanjing, China

**Keywords:** large-panicle japonica rice, panicle branches, vascular bundle structures, inferior spikelets, grain filling

## Abstract

The vascular bundles of rice panicles serve to connect the source and the sink, as well as serving as a channel for the transportation of materials. In this study, two homozygous japonica rice strains were used as materials. The vascular bundle structures of the branches in different positions within a rice panicle were observed, and their cross-sectional areas were calculated. In addition, the ultrastructure of the central large vascular bundle (LVB) phloem in the rachillae of superior spikelets (SS) and inferior spikelets (IS) was observed during the grain filling period. Moreover, the soluble sugar and protein contents of the SS and IS rachillae were also measured to study whether the differences in the structure of vascular bundles of the branches were related to the plumpness of grain at different positions. The results showed that vascular bundle cross-sectional areas of the basal primary branches were greater than those in the upper primary branches. Moreover, there was little difference in the areas of vascular bundles between the basal secondary branches and upper secondary branches. However, the vascular bundle areas of the IS rachillae were lower than those in the SS rachillae. Therefore, we believe that the poor vascular tissue channel of the IS rachillae could be the limiting factor in IS plumpness. The results also showed that a similar time course in the degradation pattern of some organelles of the sieve elements and companion cells in central LVB was observed in the SS rachillae and IS rachillae during the grain filling period. Compared with the IS rachillae, more abundant mitochondria and plasmodesmata were found in the companion cells of SS rachillae at the beginning of the filling stage, while no significant differences between SS and IS rachillae were identified at the middle and late filling stages, which implies that the SS rachillae were relatively more effective at transportation compared with the IS rachillae at the initial filling stage. Therefore, the undeveloped vascular bundles of the IS rachillae and their poor physiology and lack of ability to transport at the initial filling stages could be the limiting factor in IS plumpness.

## Introduction

Rice (*Oryza sativa* L.) is one of the most important food crops in the world. Due to the continuing growth of the global population and a decrease in arable land, increasing the rice yield per unit area is the main goal of breeders (Khush, 2005). In rice breeding, the sink capacity has been expanded by increasing the number of spikelets per panicle and creating large-panicle rice varieties (Kato et al., 2007). However, these varieties generally do not produce the yield expected due to the low seed setting rate and grain weight of inferior spikelets (Zhang et al., 2009; Yang and Zhang, 2010). In rice plants, spikelets are divided into superior spikelets (SS) and inferior spikelets (IS) based on their location on the branch and time of flowering (Ishimaru et al., 2003). Typically, the SS are located on upper primary branches, and they flower earlier, fill more quickly, and produce larger and heavier grains. In contrast, the IS are located on the basal secondary branches, and they flower later, fill more slowly, and produce smaller grains (Mohapatra et al., 1993; Ishimaru et al., 2003). Therefore, improving IS grain filling is of substantial significance to ensure high-yield potential for large-panicle rice varieties.

The reasons for poor IS grain filling have been studied in-depth from the following aspects, including assimilation supply (Tang et al., 2009; Yang and Zhang, 2010; You et al., 2017), hormone balance (Zhang et al., 2009; Zhu et al., 2011; Wang et al., 2015), enzyme activity (Tang et al., 2009; Wang et al., 2015), and gene expression (Mohapatra and Panigrahi, 2011; You et al., 2016). However, whether the vascular bundle structure of panicle branches is a crucial factor for poor IS grain filling remains unclear.

The vascular bundle is the transportation system that links the source to the sink and strongly affects the transport efficiency of photosynthetic assimilates (Peterson et al., 1982). There is a significant positive correlation between grain yield and the number of vascular bundles in rice (Mohammad et al., 1994), wheat (Evans et al., 1970), and oats (Peterson et al., 1982). In a study of the relationship between vascular bundles structure of the panicle and grain filling, Ma et al. (2002) found that, in the panicle neck nodes of rice, the ratio of the area of culm wall to cross section of culm significantly positively correlated with the percentage of grain filling. Ming et al. (2004) found that the undeveloped vascular bundles in the low position of the panicle could be an important reason for poor IS filling. Huang et al. (2004) also came to a similar conclusion. They observed that compared with the primary branches with SS in the middle and upper positions of the panicle, the vascular bundles of the secondary branches at the basal position of the panicle with IS were poorly developed, which mainly manifested in smaller vessel and phloem areas of the vascular bundles. This phenomenon is likely to cause the poor transportation of photosynthetic products supplied to the IS. However, Li M. Y. et al. (2000) concluded that the obstacles to the seed setting in indica-japonica hybrid combinations were not caused by the differences in panicle anatomical structures. Previous studies primarily focused on comparisons of the vascular bundle area between the primary and secondary branches on the upper and lower positions of the panicle. However, there are few studies that directly compare the structure of rachillae in SS and IS. Moreover, it is rarer to observe the changes in the ultrastructure of the phloem sieve elements and companion cells in the central vascular bundle of the rachillae during the grain filling period. These results collectively demonstrate that there is still a lack of comprehensive research on the relationship between vascular bundle structures of rice panicle branches and IS grain filling.

This study investigated whether the differences in the structure of vascular bundles of the branches were related to the plumpness of grain at different panicle positions in japonica rice with large panicles. The vascular bundle structures of the branches in different positions of the panicle were observed, and the cross-sectional areas of vessel and phloem were also calculated using an optical microscope and a scanning electron microscope. In addition, the ultrastructure of central large vascular bundle phloem in the SS and IS rachillae was observed during the grain filling period using transmission electron microscopy. Moreover, the physiologically active of SS and IS rachillae were measured, and we then analyzed the relationship between vascular bundles structure of the branches and grain development to reveal the underlying causes of differences in grain filling between the SS and IS. The studies described above are expected to provide a theoretical basis to improve IS grain filling.

## Materials and Methods

### Plant Materials

The two homozygous large-panicle japonica rice strains W1844 and WJ165 that were selected for the experiment were provided from the State Key Laboratory of Rice Genetics and Germplasm Innovation in Nanjing Agricultural University, Nanjing, China. W1844 and WJ165 are the intermediate materials of breeding, but their genetic characteristics have stabilized. They have more than 250 grains per panicle and thus are typical of large-panicle rice varieties. In addition, they have significant differences in filling between SS and IS (You et al., 2016).

### Experimental Design

This experiment was conducted at the Danyang Experimental Base of the Nanjing Agricultural University, Jiangsu Province, China (31°54′31″N, 119°28′21″E) during the rice-growing season in 2016. The seedlings were sown on 28 May and transplanted on 18 June at a hill that was spaced 13.3 cm × 30 cm with three seedlings per hill. The plot dimensions were 5 m × 10 m. Each rice strain was grown in three replicate plots in a completely randomized block design. The soil at the experimental site was clay loam. The amounts of urea, Ca(H_2_PO_4_)_2,_ and KCl applied throughout the whole growing season were 600 kg ha^–1^, 231 kg⋅ha^–1^, and 295 kg⋅ha^–1^, respectively. Among them, urea was applied at the ratio of base fertilizer: tillering fertilizer: panicle fertilizer = 4:2:4, Ca(H_2_PO_4_)_2_ was consistently used as the base fertilizer, and KCl was applied at a ratio of base fertilizer: panicle fertilizer = 5:5. The base fertilizer was applied before transplanting; the tiller fertilizer was applied 7 days after transplanting, and the panicle fertilizer was applied when the leaf-age remainder was 3.5. Cultivation and management measures were applied based on the technical requirements of the local field.

### Sampling and Measurement

**FIGURE 1:**
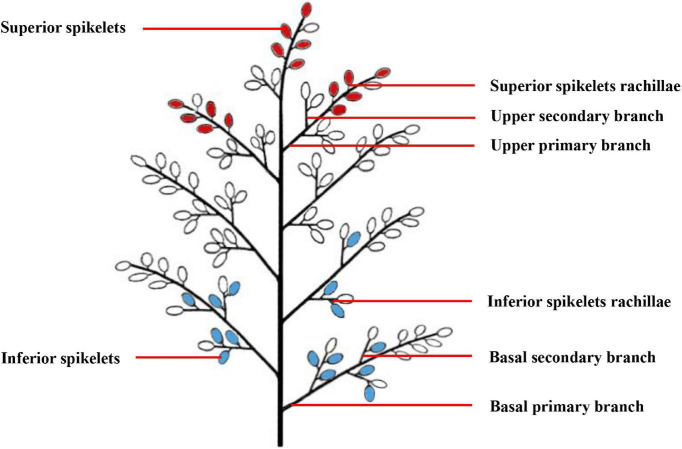
A pattern diag ram of rice panicle structure. The red grains represent the superior spikelets, and the blue grains represent the inferior spikelets.

A total of 1,000 single stems with similar growth patterns that flowered on the same day were labeled during the heading-blooming stage. We sampled 100 tagged panicles from each plot every 5 days from anthesis to maturity. The sampling sites included the upper primary branch, upper secondary branch, SS rachillae, basal primary branch, and basal secondary branch, as well as the IS rachillae. The specific sampling location is shown in Figure 1. From the junction of the primary branch and the cob, the primary branch about 1-mm long was cut off and used as a sample of the upper primary branch or the basal primary branch. From the junction of the secondary branch and the primary branch, the secondary branch about 1-mm long was cut off and used as a sample of the upper secondary branch or the basal secondary branch. The branches directly connected to the SS and IS were cut into about 1-mm small pieces to be used as samples of SS rachillae or IS rachillae. To observe the structure of vascular bundles in the branches in different positions of the panicle, one-fifth of the branches sampled were fixed in FAA solution (38% formaldehyde: glacial acetic acid: 70% alcohol = 1:1:18), and then stored at 4°C for optical microscopy (OM) observation. Another one-fifth of the branches sampled were fixed in a solution of 2.5% glutaraldehyde [0.1-M phosphate-buffered saline (PBS), pH 7.2] for 2 h at room temperature and then stored at 4°C for observation with scanning electron microscopy (SEM) and transmission electron microscopy (TEM). The remaining branch samples were frozen in liquid nitrogen and stored at −80°C to determine the physiological activity of branches. The SS were considered to be the grains on the three primary branches on the upper part of the panicle, while the IS were considered to be the grains on three primary branches in the basal part of the panicle.

### Observation of Vascular Bundle Structures in Panicle Branches

#### Optical Microscopy Observations

The vascular bundle structure of the panicle branches was observed using slices combined with phloroglucinol staining. First, branches were removed from the FAA solution and cut into thin slices with a freezing microtome (NX50; ThermoFisher Scientific, Waltham, MA, United States), which was used to cut the branches as thinly as possible while maintaining the integrity of the tissue, and the slices were then placed on the slide. One gram of phloroglucinol was dissolved in 100 ml of 70% ethanol. One drop of the phloroglucinol solution was added first, after 30 s of reaction, one drop of 18% HCl solution was added. The mixture was then covered with a cover glass and observed under a 10 × optical microscope (BX53; Olympus, Tokyo, Japan). The samples of branches or rachillae come from 15 tagged panicles. We primarily observed the structure of vascular bundles and counted the number of large vascular bundles (LVB) and small vascular bundles (SVB). Simultaneously, the Olympus software that was provided with the OM was used to measure the cross-sectional area of each vascular tissue, including the vessel area and the phloem area of LVB and SVB, total areas of vessels, total areas of phloem, as well as the total areas of vascular bundles.

#### Scanning Electron Microscopy Observations

The samples were removed from the 2.5% glutaraldehyde solution (0.1-M PBS, pH 7.2) and washed three times with PBS before dehydration using a gradient concentration of ethanol for 30 min each time. The ethanol was replaced by isoamyl acetate and dried at the critical point of CO_2_. The samples were affixed to the copper sample table using double-sided tape, and the section was gold-plated with sputter coater (JFC-1600; Japan). Finally, the samples were observed using SEM (S-3000 N; Hitachi, Tokyo, Japan). We focused on comparing the structure of xylem and phloem of vascular bundles in the panicle branches.

#### Transmission Electron Microscopy Observations

The samples were removed from the 2.5% glutaraldehyde solution, rinsed three times with a 0.1-M solution of phosphoric acid, fixed with 1% osmic acid solution for 2–3 h, and rinsed again. The rinsed samples were dehydrated using a gradient concentration of ethanol for 15–20 min for each gradient and slowly infiltrated with pure acetone and Epon 812 resin (2:1) at room temperature for 3–4 h, and then placed in Epon 812 resin for embedding. The samples were first incubated overnight in a 37°C oven, incubated for 12 h in a 45°C oven, finally, incubated for 24 h in a 60°C oven for curing. An ultramicrotome (LKB-I; Sweden) was used to slice the samples 50–60-nm thick, and they were then stained with 3% uranyl acetate-lead citrate. Finally, the slices were observed and filmed while under TEM (JEM-1200EX; Japan). The developmental process of the phloem in SS rachillae and IS rachillae at different days after anthesis was emphatically observed. Both the SEM and TEM experiments were conducted in the Life Science Experimental Center of Nanjing Agricultural University.

### The Physiological Activity of Superior Spikelets Rachillae and Inferior Spikelets Rachillae

#### Determination of Protein Content

The content of protein was determined as described by Li H. S. et al. (2000). The fresh sample (0.5 g) was cut and mixed and placed into a mortar with 2 ml of distilled water; the samples were grounded into a homogenate and transferred to a 10-ml centrifuge tube. The mortar was washed several times with 6 ml of distilled water, and the washing liquid was collected in the same centrifuge tube and left it for 30 min to fully extract. The centrifuge tube was centrifuged at 4,000 rpm for 30 min, the precipitate was discarded, and the supernatant was transferred to a 10-ml volumetric flask, and the volume was fixed with distilled water. Took a new 10 mL graduated glass test tube with stopper, 0.1 mL of the extract was pipetted, and put it into a new 10 mL graduated glass test tube with stopper, then added 5 mL of Coomassie Brilliant Blue G-250, and mixed them. After standing for 2 min, colorimetric determination was performed at 595 nm.

#### Determination of Soluble Sugar Content

The soluble sugar content determination method was modified from the method of Zhang (1992). The fresh sample (50 mg) that was cut and mixed and placed into a 10-ml centrifuge tube with 4 ml of distilled water, and the samples were placed into an 80°C water bath for 40 min. After cooling, the sample was centrifuged at 3,000 rpm for 30 min. The extraction process was repeated two times, and the supernatants were combined and distilled water was added up to 10 ml. 1 mL of the extract and 5 mL of anthrone reagent [150 mg of anthrone dissolved in 100 mL of dilute sulfuric acid (76 ml of concentrated sulfuric acid plus 30 ml of distilled water)] was added to a 10 mL centrifuge tube, and then placed the centrifuge tube into a boiling water bath for 20 min. After cooling, colorimetric determination was performed at 620 nm.

### Assessment of Panicle Characteristics

At maturity, approximately 90 tagged panicles from each rice strain were harvested. The samples were naturally dried; the grains per panicle, plumpness, as well as the grain weight and the seed setting rate of SS and IS, were measured. From all the grains of 90 panicles, 100 grains were randomly selected to determine the grain length and grain width with a Vernier caliper. The seed setting rate was determined as described by Kobata et al. (2010). The plumpness of grain = weight of fertilized grain/weight of the fully filled grain, and the plumpness of grain was determined as described by Zhu et al. (1995).

### Statistical Analysis

At least three biological replicates were used for each treatment and control. The data were analyzed statistically using standard ANOVA, and the mean values were tested by the least significant difference (LSD) test at the 5% level using SPSS16.0 (SPSS, Inc., Chicago, IL, United States).

## Results and Analysis

### Grain Weight and the Seed Setting Rate

Table 1 demonstrates the grains per panicle, plumpness, the grain weight, the seed setting rate, and grain size of the rice strains. Both W1844 and WJ165 have more than 250 spikelets per panicle, so they are both typical large panicle rice strains. The grain weight and the seed setting rate of IS in W1844 and WJ165 were significantly lower than those of SS, and the grain weight of IS was 21.45 and 9.60% lower than that of SS, respectively, while the seed setting rate of IS was 11.74 and 7.17% lower than that of SS, respectively, indicating that there were significant differences in the grain filling and seed setting between SS and IS in the two rice strains. WJ165 had higher grain length, grain width, and grain weight compared with W1844, whereas the plumpness and the seed setting rate were lower than that of W1844, indicating that WJ165 has larger grains but poorer plumpness compared with W1844.

**TABLE 1:** Grain weight and the seed setting rate of the test materials.

Materials	Grains per panicle	Grain length (mm)	Grain width (mm)	Plumpness (%)	Grain weight (mg/grain)		Seed setting rate (%)	
					Superior	Inferior	Superior	Inferior
W1844	265.0 ± 7.6	7.10 ± 0.3	3.46 ± 0.1	77.0 ± 1.0	26.6 ± 0.1 a	20.9 ± 0.4 b	97.1 ± 0.5 a	85.7 ± 0.3 b
WJ165	257.3 ± 2.5	8.22 ± 0.2	3.76 ± 0.1	70.7 ± 0.5	32.3 ± 0.1 a	29.2 ± 0.1 b	90.6 ± 0.5 a	84.1 ± 0.5 b

*Different lowercase letters labeled after the data from the same character under the same variety indicate significant differences at the 0.05 level.*

### Structural Differences in the Vascular Bundles

**FIGURE 2:**
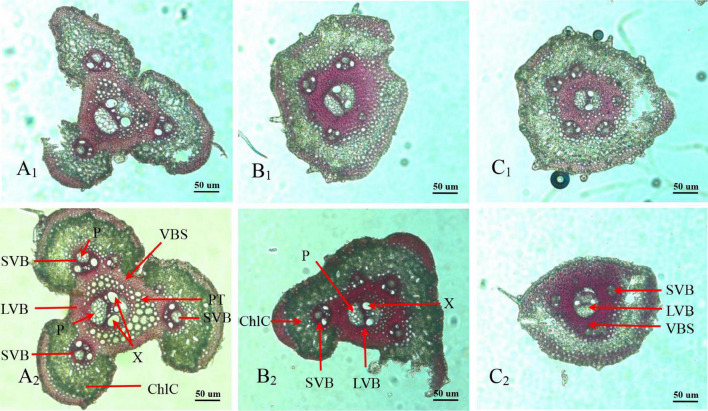
Microscopical structure observation of vascular bundles between different positional rachillae in W165. Groups **(A_1_,B_1_,C_1_)** are the upper primary branch, upper secondary branch, and superior spikelets rachillae, respectively, in the WJ165; Groups **(A_2_,B_2_,C_2_)** are the basal primary branch, basal secondary branch, and inferior spikelets rachillae, respectively, in the WJ165; LVB, large vascular bundle; SVB, small vascular bundle; X, xylem; P, phloem; VBS, vascular bundle sheath; ChlC, chlorenchyma cell; PT, parenchymatous tissue. All the pictures were taken using an optical microscope (×10).

**FIGURE 3:**
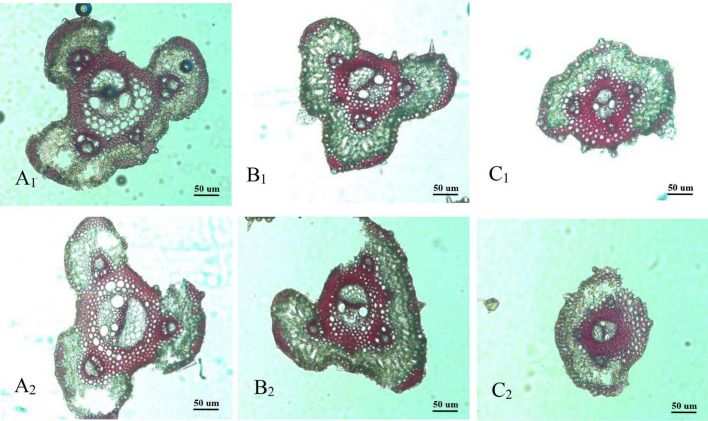
Microscopical structure observation of vascular bundles between different positional rachillae in W1844. Groups **(A_1_,B_1_,C_1_)** are the upper primary branch, upper secondary branch, and superior spikelets rachillae, respectively, in the W1844; Groups **(A_2_,B_2_,C_2_)** are the basal primary branch, basal secondary branch, and inferior spikelets rachillae, respectively, in the W1844; all the pictures were taken using an optical microscope (×10).

Panicle branches are composed of an epidermis, parenchyma cells (PC), sclerenchymatous cells (SC), and vascular bundles. Among them, the vascular bundles typically include a central large vascular bundle (LVB) and 3–5 small vascular bundles (SVB) (Chaudhry and Nagato, 1970; Jin et al., 1999). The vascular bundle structures of the panicle branches in WJ165 and W1844 were observed under an optical microscope (OM) at 15 days post anthesis (DPA) as shown in Figures 2, 3. It is apparent from the figures that both LVB and SVB on the primary branches have clear vessels and sieve tubes. Compared with the primary branches, the cross-sectional area of each vascular bundle was smaller on the secondary branches, while they were the smallest in the SS rachillae and IS rachillae. A comparison of the branches of different parts in the rice panicle indicated that the phloem areas of central LVB in the basal primary branch were larger than those of the upper primary branches (Figures 2A_1_,A_2_, 3A_1_,A_2_). In contrast, the LVB and SVB of the basal and upper secondary branches displayed a similar developmental status (Figures 2B_1_,B_2_, 3B_1_B_2_).

A comparison of the structure of vascular bundles in the SS rachillae and IS rachillae indicated that the vessels and sieve tubes of LVB and SVB in the IS rachillae were smaller than those of the SS rachillae (Figures 2C_1_,C_2_, 3C_1_,C_2_). Therefore, the vascular bundle cross-sectional areas of the basal primary branches were greater than those in the upper primary branches. Moreover, there was little difference in the areas of vascular bundles between the basal secondary branches and upper secondary branches. However, the vascular bundle areas of the IS rachillae were lower than those in the SS rachillae.

**FIGURE 4:**
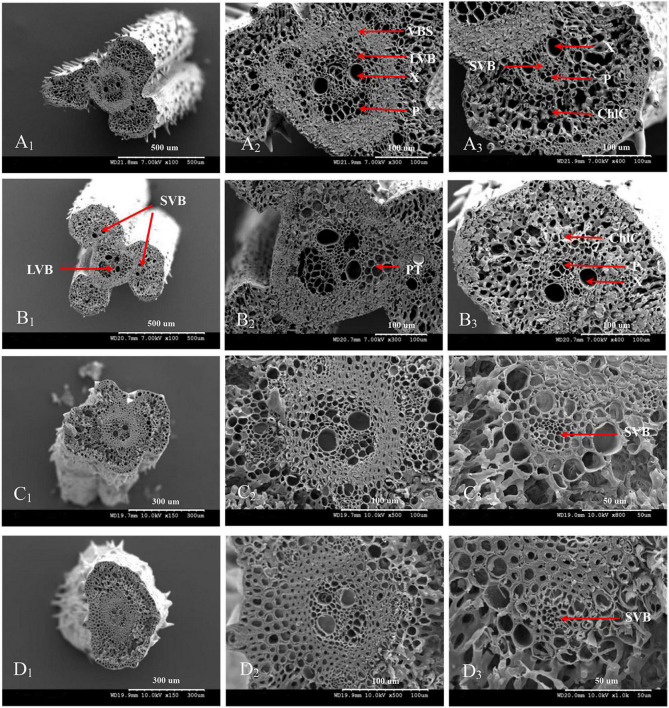
Microscopical structure observation of the vascular bundles between different positional rachillae in WJ165. Groups **(A,B)** are cross sections of the upper and basal primary branches in WJ165. Groups **(A_1_,B_1_)** are the entire cross section of the branches (×100); Groups **(A_2_,B_2_)** are the central large vascular bundles (× 300); Groups **(A_3_,B_3_)** are the small vascular bundles (×300). Groups **(C,D)** are the cross section of the upper-superior spikelets rachillae and the basal-inferior spikelets rachillae in WJ165. Groups **(C_1_,D_1_)** are the entire cross section of the branches (×150); Groups **(C_2_,D_2_)** are the central large vascular bundles (×500), and Groups **(C_3_,D_3_)** are the small vascular bundles (×1,000). LVB, large vascular bundle; SVB, small vascular bundle; X, xylem; P, phloem; VBS, vascular bundle sheath; ChlC, chlorenchyma cell; PT, parenchymatous tissue.

**FIGURE 5:**
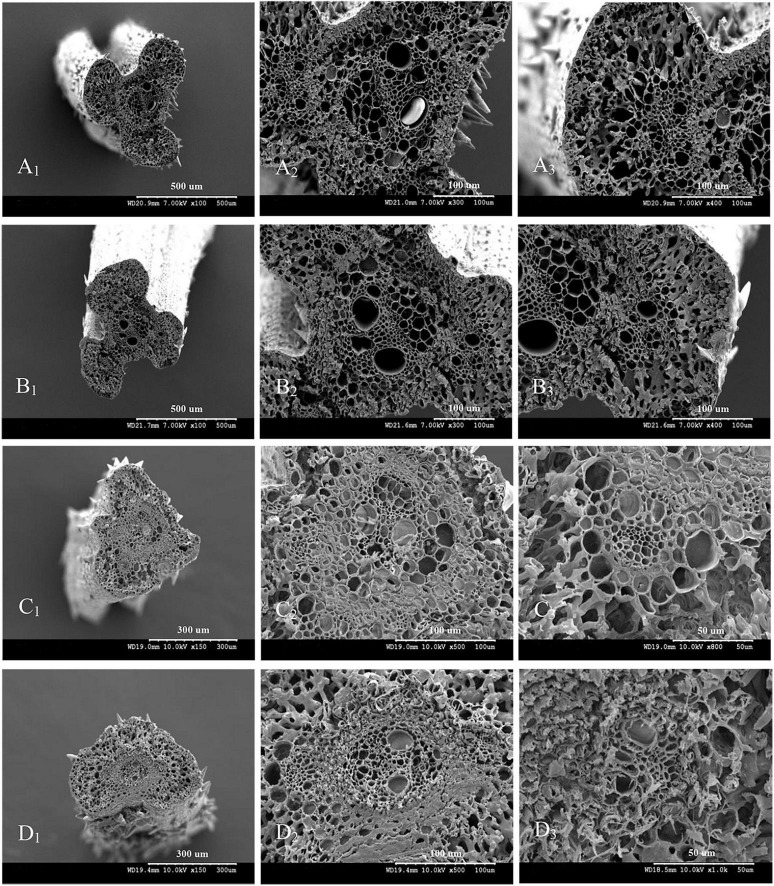
Microscopical structure observation of the vascular bundles between different positional rachillae in W1844. Groups **(A,B)** are cross sections of the upper and basal primary branches in W1844. Groups **(A_1_,B_1_)** are the entire cross sections of the branches (×100); Groups **(A_2_,B_2_)** are the central large vascular bundles (×300); Groups **(A_3_,B_3_)** are the small vascular bundles (×300). Groups **(C,D)** are cross sections of the upper-superior spikelets rachillae and the basal-inferior spikelets rachillae in W1844. Groups **(C_1_,D_1_)** are the entire cross sections of the branch (×150); Groups **(C_2_,D_2_)** are the central large vascular bundles (×500); Groups **(C_3_,D_3_)** are the small vascular bundles (×1,000).

The vascular bundle structures of the panicle branches in WJ165 and W1844 when observed under a scanning electron microscope (SEM) at 15 DPA are shown in Figures 4, 5. The figures showed that both LVB and SVB on the upper and basal primary branches have clearly visible vessels and sieve tubes, and their cross-sectional areas were relatively large. However, the vessels and sieve tubes of the vascular bundles in the SS and IS rachillae were not only poorly developed but also had a smaller cross-sectional area. In particular, the IS rachillae were the most poorly developed. The results of SEM observation were the same as those of OM, that is, the vascular bundles of the IS rachillae were poorly developed and differentiated.

### The Cross-Sectional Areas of Vascular Bundles in the Panicle Branches

The cross-sectional areas of vascular bundles in the panicle branches at 15 DPA are shown in Tables 2, 3. It is apparent from Table 2 that the number and the vessel areas of the LVB in basal primary branches did not differ from those of the upper primary branches, while the areas of the phloem were significantly higher than those of the upper primary branches. However, the number and phloem areas of the SVB in basal primary branches did not differ from those of the upper primary branches, while the areas of vessels were significantly lower than those of the upper primary branches. In general, compared with the upper primary branches, although the basal primary branches have a lower total area of vessels, they had higher total areas of the sieve tubes. In addition, the total areas of vascular bundles were also higher. These results indicate that the vascular tissue channels of the basal primary branches are more developed than those of the upper primary branches.

**TABLE 2:** Comparison of vascular bundle areas in W1844 panicle branches.

Position	Large vascular bundle			Small vascular bundle			Total areas of vessel (μ m^2^)	Total areas of phloem (μ m^2^)	Total areas of vascular bundle (μ m^2^)
	Numbers	Areas of vessel (μ m^2^)	Areas of phloem (μ m^2^)	Numbers	Areas of vessel (μ m^2^)	Areas of phloem (μ m^2^)			
Upper primary branch	1.13 ab	2,680 a	6,194 b	3.33 bc	1,479 a	2,445 a	4,159 a	8,639 b	12,798 b
Upper secondary branch	1.00 b	1,100 b	2,718 c	3.00 c	762 c	1,235 b	1,862 c	3,953 c	5,815 c
Upper SS rachillae	1.00 b	877 bc	1,970 d	3.75 a	820 c	1,230 b	1,697 c	3,200 d	4,897 d

Basal primary branch	1.20 a	2,763 a	7,754 a	3.07 c	1,129 b	2,455 a	3,892 b	10,209 a	14,101 a
Basal secondary branch	1.00 b	985 bc	2,683 c	3.62 ab	834 c	1,050 b	1,819 c	3,733 cd	5,553 cd
Basal IS rachillae	1.00 b	843 c	1,586 d	3.30 bc	798 c	856 c	1,641 c	2,442 e	4,083 e

*Different small letters in the same line indicate a significant difference at the 0.05 level among the vascular bundles.*

**TABLE 3:** Comparison of vascular bundle areas in WJ165 panicle branches.

Position	Large vascular bundle			Small vascular bundle			Total areas of vessel (μ m^2^)	Total areas of phloem (μ m^2^)	Total areas of vascular bundle (μ m^2^)
	Numbers	Areas of vessel (μ m^2^)	Areas of phloem (μ m^2^)	Numbers	Areas of vessel (μ m^2^)	Areas of phloem (μ m^2^)			
Upper primary branch	1.00 b	2,199 b	5,338 b	4.00 bc	2,117 b	3,402 b	4,316 b	8,740 b	13,056 b
Upper secondary branch	1.00 b	818 cd	2,576 cd	4.33 ab	1,393 cd	1,504 c	2,211 c	4,080 cd	6,291 cd
Upper SS rachillae	1.00 b	732 cd	1,915 de	4.78 a	1,514 c	1,667 c	2,246 c	3,582 de	5,828 d

Basal primary branch	1.57 a	3,528 a	8,255 a	4.00 bc	2,539 a	3,704 a	6,067 a	11,959 a	18,026 a
Basal secondary branch	1.20 b	1,261 c	3,208 c	3.50 c	1,215 d	1,622 c	2,476 c	4,830 c	7,306 c
Basal IS rachillae	1.00 b	598 d	1,412 e	4.00 bc	781 e	1,220 d	1,379 d	2,632 e	4,011 e

*Different small letters in the same line indicate a significant difference at the 0.05 level among the vascular bundles.*

A comparison of the upper secondary branches and basal secondary branches of W1844 indicated that the vessel and phloem areas of LVB and SVB in the basal secondary branches were slightly lower than those of the upper secondary branches. Therefore, the total vessel and phloem areas of the vascular bundles in the basal secondary branches were also slightly lower than those of the upper secondary branches, but there was no significant difference. In short, the development of the vascular tissue in basal secondary branches was similar to that of the upper secondary branches.

A comparison of the SS rachillae and IS rachillae of W1844 indicated that the number and the phloem areas in the SVB of IS rachillae were significantly lower than those of the SS rachillae, while the areas of other vascular tissues did not differ significantly from those of SS rachillae. Therefore, the total areas of phloem and the total areas of vascular bundles in the IS rachillae were significantly lower than those of the SS rachillae, which showed that the IS rachillae vascular tissue was more poorly developed than that of the SS rachillae.

In WJ165, the changes in cross-sectional areas of the vascular bundles in the panicle branches were similar to those of W1844 (Table 3). The performance of total vessel areas, total phloem areas, and total vascular bundle areas were as follows: basal primary branches > upper primary branches; basal secondary branches≈upper secondary branches; and IS rachillae<SS rachillae.

### The Ultrastructure of the Phloem in Central Vascular Bundles of the Superior Spikelets Rachillae and Inferior Spikelets Rachillae

**FIGURE 6:**
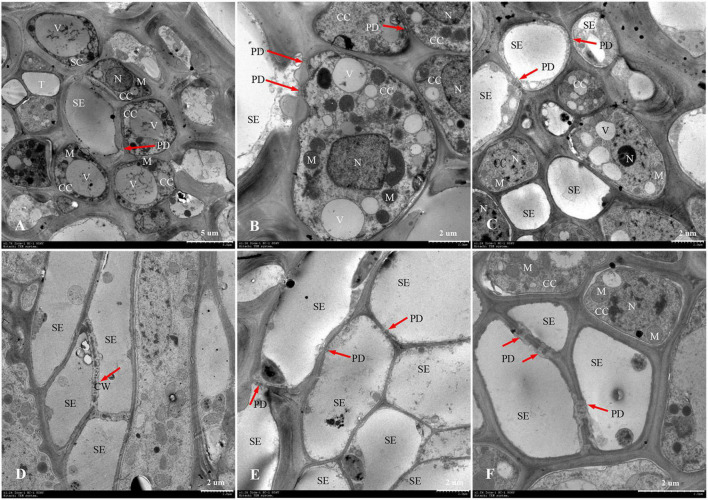
Developmental changes of sieve elements and companion cells at central vascular phloem for different positional rachillae. **(A)** SE and CC structure of SS rachillae at 5 DPA (×700), the arrow indicates PD; **(B)** CC structure of SS rachillae at 5 DPA (×1,500), showing PD between the SEs and CCs, as well as PD between the CCs; **(C)** SE and CC structure of SS rachillae at 10 DPA (×1,200), showing the degradation of nucleus and vacuole in the CC; **(D)** SE structure of SS rachillae at 15 DPA (×1,200), showing the degradation of cell wall; **(E)** SE and CC structure of SS rachillae at 20 DPA (×1,200); **(F)** SE and CC structure of IS rachillae at 20 DPA (×2,500). SE: sieve element; CC: companion cell; DPA: days post anthesis; PC: parenchyma cell; SC: sclerenchymatous cell; PD: plasmodesmata; V: vacuole; M: mitochondrion; T: tube; N: nucleus; CW: cell wall.

**FIGURE 7:**
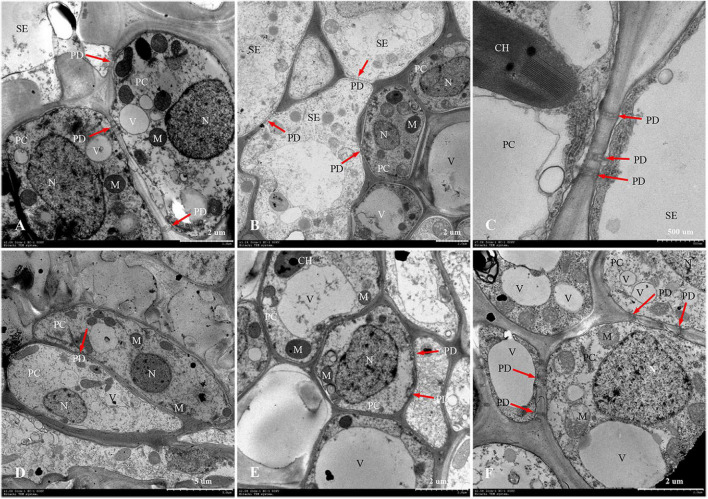
Developmental changes of parenchyma cells at central vascular phloem for different positional rachillae. **(A)** PC of SS rachillae at 5 DPA (×2,000), the arrow indicates PD; **(B)** PC of IS rachillae at 5 DPA (×1,200), showing organelles; **(C)** SE and PC structure of SS rachillae at 5 DPA (×7,000), showing the structure of chloroplast and PD; **(D)** PC of SS rachillae at 20 DPA (×1,000), showing the degradation of organelles; **(E)** PC of IS rachillae at 20 DPA (×2,000); **(F)** PC of IS rachillae at 30 DPA (×2,500), showing degradation of the vacuole, mitochondria and nucleus. SE: sieve element; PC: parenchyma cell; DPA: days post anthesis; PD: plasmodesmata; V: vacuole; M: mitochondrion; N: nucleus; CH: chloroplast.

The phloem of the central vascular bundles in the rachilla is composed of sieve elements (SEs), companion cells (CCs), and phloem parenchyma cells (PCs) (Wang et al., 1994). Among them, the SEs and CCs are located in the center of the phloem, whereas the PCs are primarily distributed around the phloem. The ultrastructure of SS rachillae and IS rachillae vascular tissues in W1844 during the grain filling process when viewed using TEM are shown in Figure 6. At 5 DPA, the SEs of SS rachillae had signs of deterioration. For example, the nucleus had disintegrated and disappeared, and the cytoplasm had agglutinated and gathered near the cell wall (Figure 6A). At this time, the CCs near the SEs have denser cytoplasm with abundant organelles and numerous small vacuoles. Moreover, a large number of plasmodesmata (PDs) were also found between the SEs and CCs (Figure 6B). In addition, there were a large number of PDs between the CCs, PCs, as well as the SEs and PCs in the SS rachillae during this period (Figures 6B, 7A,C).

At 10 DPA, various organelles in the CCs of SS rachillae began to show signs of degeneration. It was apparent that the central vacuoles degraded into several small vacuoles. Moreover, in some CCs, only nucleoli were observed. In addition, the number of mitochondria was also reduced (Figure 6C). At 15 DPA, the SEs of SS rachillae have basically matured, and the cell wall between the SEs began to dissolve and shrink (Figure 6D). At 20 DPA, most of the organelles in the SEs of SS rachillae had completely disintegrated (Figure 6E). Moreover, in the CCs of the IS rachillae, the nuclei, small vacuoles, and mitochondria (M) still can be seen. However, the deterioration of the most organelles was visible (Figure 6F).

The phenomenon described above indicated that the SEs of SS rachillae and IS rachillae may be fully differentiated before flowering and have begun to mature at the early stage of filling. They matured in 15–20 DPA and became a hollow structure. However, during the initial stage of filling, the CCs still maintained a relatively complete cell structure. They then began to deteriorate during the filling process, and this degeneration was particularly clear in the early and middle period of filling. These results indicate that, compared with the SS rachillae, the deterioration of CCs in the IS rachillae occurred later.

In W1844, the ultrastructure of parenchyma cells in the rachillae phloem had clear dynamic changes during the filling process (Figure 7). At 5 DPA, the PCs of SS rachillae were clear and fully differentiated, with dense protoplasm, large nuclei and vacuoles, chloroplasts, and many spherical or elliptical mitochondria. Moreover, there were abundant PD between the PCs and between SEs and PCs (Figures 7A,C). Moreover, the PCs of IS rachillae were also intact and contained large nuclei and vacuoles, as well as thin cytoplasm. There were mitochondria in these PCs, but there were fewer than those of the SS rachillae (Figure 7B). At 20 DPA, there were still larger nuclei in the PCs of SS rachillae, but the mitochondria gradually disintegrated; the morphology became obscured, and the numbers were significantly reduced. In addition, there were some small vacuoles scattered in the PCs (Figure 7D). Moreover, some large nuclei and intact large vacuoles, as well as mitochondria, could be observed in the PCs of IS rachillae (Figure 7E). At 30 DPA, the organelles of PCs in IS rachillae clearly degraded; the nucleus completely disintegrated, and there were only a few mitochondria that remained in the PCs (Figure 7F). The phenomenon described above indicates that, in the vascular tissue of rice rachillae, the deterioration of PCs and the disintegration of various organelles in the PCs occurred significantly later than the disintegration of CCs. This could enable the transfusion tissue to function in rice rachillae during the middle and late stages of rice filling.

### Changes in the Soluble Sugar and Protein Contents of Both Superior Spikelets Rachillae and Inferior Spikelets Rachillae

**FIGURE 8:**
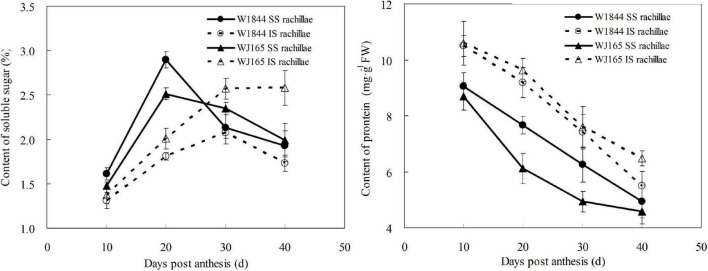
Changes in the soluble sugar and protein contents of both SS rachillae and IS rachillae from strains W1844 and WJ165 during grain filling. Vertical bars represent mean values ± SE (*n* ≥ 4).

Figure 8 illustrates changes in the contents of soluble sugar and proteins of both the SS rachillae and IS rachillae from strains W1844 and WJ165 during grain filling. During the early filling stage, the content of soluble sugar in the SS rachillae was significantly higher than that of IS rachillae, reached its highest value after 20 DPA, and then decreased. In contrast, in the IS rachillae, the soluble sugar content was very low during the early filling stage and increased slowly after 10 DPA, peaking at 30 DPA.

As shown in Figure 8, the protein content of SS rachillae and IS rachillae was the highest at 10 DPA and then continued to decline with the filling process. Moreover, the protein content of SS rachillae was always lower than that of IS rachillae during the whole filling process. The changes of soluble sugar and protein content in SS rachillae and IS rachillae were consistent in strains W1844 and WJ165.

## Discussion

### Physiological Differences Between Superior Spikelets and Inferior Spikelets of W1844 and WJ165

The phenomena of poor plumpness and low seed setting rates are common in large-panicle rice varieties, and this is primarily owing to poor IS grain filling and the formation of empty and blighted grains of rice (Yang and Zhang, 2006). These phenomena were also observed in this study. The grain weight and the seed setting rate of IS in W1844 and WJ165 were significantly lower than those of the SS (Table 1). Some studies have concluded that this is due to the competition for photosynthetic assimilates between SS and IS, but the IS are at a disadvantage (Duan et al., 1999; Liu et al., 2021). Mohapatra et al. (1993) and Xu et al. (1998) also found that there is a competitive effect of assimilation products between different grains on the same panicle, and they concluded that one of the important factors that affect the distribution of assimilates, grain plumpness, and seed setting characteristics is the vascular bundles structure of the branches in different positions of the panicle. The number and cross-sectional areas of the vascular bundles in the panicle branches positively correlated with grain plumpness and grain weight (Ming et al., 2004; Huang et al., 2005).

Plumpness is the most effective indicator to use to measure the fullness of rice grains (Zhu et al., 1995). In this study, the grain weight and plumpness of the two rice strains were substantially different. In particular, WJ165 demonstrated higher grain length, grain width, and grain weight compared with W1844, whereas the plumpness and the seed setting rate were lower than those of W1844, indicating that WJ165 has larger grains but poorer plumpness compared with W1844 (Table 1). The way of photosynthetic assimilates entering into caryopsis was as follows: center vascular of rachilla—vascular of ovary back—nucellar projection—nucellus layer apoplast—endosperm (Wang et al., 1995). Compared with W1844, the grains of WJ165 are larger, and there is a relatively longer distance between the vascular system of ovary back and the endosperm. Therefore, there is resistance during the process of conveying the photosynthetic assimilates to the endosperm, and this may be one of the reasons why the grain plumpness of WJ165 is lower than that of W1844.

### Differences in the Structure and the Cross-Sectional Area of Branch Vascular Bundles in Different Parts of the Panicle

The panicle branches are the places where rice grains grow, and they are also the channels for nutrients to enter the grains. The development of vascular bundles directly affects the transportation of inorganic and organic matter to the grain, which, in turn, determines the development of grain. Previous studies have shown that the structure of vascular bundles in rice panicles is closely related to grain plumpness. For example, Huang et al. (2004) found that, compared with the primary branches with SS in the middle and upper positions of the panicle, the vascular bundles of the secondary branches at the basal position of the panicle with IS were poorly developed. This primarily manifested in the smaller vessel and the phloem area of the vascular bundles. Ming et al. (2004) also hypothesized that the undeveloped vascular bundles in the low position of the panicle could be an important reason for poor IS filling. The results of this study showed similar conclusions: in W1844 and WJ165, vascular bundle cross-sectional areas of the basal primary branches were greater than those in the upper primary branches; moreover, there was little difference in the vascular bundle cross-sectional areas between the basal secondary branches and the upper secondary branches. However, the vascular bundles cross-sectional areas of the IS rachilla were lower than that in the SS rachilla. In summary, the vascular bundles of the primary and secondary branches at the base of the panicle are well-developed, whereas the vascular bundles of the IS rachilla are poorly developed. The structure proves that the vascular bundles of IS rachillae are unable to efficiently transport materials. Therefore, the underdeveloped vascular bundles of the IS rachillae could be the limiting factor of the IS filling.

### The Ultrastructural Changes in the Development of the Phloem in the Central Vascular Bundles of the Superior Spikelets Rachillae and Inferior Spikelets Rachillae

Rachillae can connect the rice grains to the panicle axis; the photosynthetic assimilates are primarily transported to the grains through the SEs of the rachillae phloem, and the transport capacity of the SEs largely depends on the CCs adjacent to them (Zhang et al., 2006). In this study, the ultrastructure of the phloem SEs and CCs in the central vascular bundle of SS rachillae and IS rachillae was observed using TEM. It was found that the CCs of the SS rachillae had numerous mitochondria (M) at the beginning of anthesis. In addition, there were abundant PD between the SEs and CCs, SEs and PCs, CCs and CCs, as well as PCs and PCs in SS rachillae during this period (Figures 6, 7). The number of M and PD in CCs can indicate the ability of SEs to transport materials. Therefore, at the beginning of the SS filling, there are a large number of M in the CCs of the phloem in SS rachillae, indicating that it is actively respiring; its energy metabolism is strong, and its SEs have a high material transport capacity, which is conducive to the filling of SS.

The ultrastructure of the phloem SEs and CCs in the central vascular bundles of SS rachillae and IS rachillae has the similar changes after the rice anthesis. The SEs of the SS rachillae have shown signs of deterioration 10 days after the rice anthesis, which could be an important sign that the SE cells have differentiated and matured and can normally perform their transport functions. At 15 days after anthesis, the SE cells of the central vascular bundle in the SS rachillae basically degenerated, and, by Day 20, after anthesis, the organelles in the CCs had also been completely disintegrated. Zhang et al. (2006) observed a similar phenomenon, and they hypothesized that this characteristic of programmed death shown by CCs may be related to the loss of phloem function. It was not until 20 days after flowering that the CCs of IS rachillae displayed obvious degeneration. In summary, this process of IS rachillae lags slightly behind that of the SS rachillae. Therefore, the contribution of the structure and physiological function of the rachillae transduction tissue to the grain filling could be mainly reflected during the early stage of filling.

### The Relationship Between the Grain Filling and the Physiological Activity of the Superior Spikelets Rachillae as Well as the Inferior Spikelets Rachillae

The protein content of rachillae is related to the development of vascular tissue. The vessels of vascular bundles are specialized dead cells that lack cytoplasm and are only composed of cell walls. Therefore, their protein content is low, and this facilitates the transportation of water and minerals. The phloem of vascular bundles is composed of SEs, CCs, and PCs (Wang et al., 1994). The SE cells have no nucleus and only part of the cytoplasm. Therefore, their protein content is also low, which aids in the transportation of organic matter. However, CCs and PCs are living cells, and they metabolize relatively vigorously. Therefore, they have a higher protein content. In this study, the protein contents of the SS rachillae and IS rachillae in rice panicles were determined. The results indicated that the protein content of the SS rachillae at each stage was lower than that of the IS rachillae, which is mainly due to the larger cross-sectional areas of vessels and SEs, and the smaller total areas of parenchyma cells in SS rachillae.

Grain filling materials originate from carbohydrates stored in the stem and sheath before heading and from photosynthetic products after heading (Liang et al., 1994; Xie et al., 2001). These assimilates are primarily transported from the source to grain in the form of sucrose. In this study, the soluble sugar content of the SS rachillae was higher than that of the IS rachillae in the early stage of grain filling (the first 20 days after anthesis), while its value was lower than that of the IS rachillae at the late stage of grain filling. This phenomenon may be due to the fact that the filling of SS had not yet reached its peak at 10 days after anthesis. In addition, the SEs of SS rachillae were not yet mature and had a small cross-sectional area; thus, there was not much sucrose flowing through the upper SS rachillae. At 10–20 days after anthesis, SS rachillae gradually matured. With the increase of the SEs cross-sectional areas and the filling intensity, the sucrose flowing through the SS rachillae also increases. However, at 20–40 days after anthesis, the soluble sugar continues to be transported to the SS through the SS rachillae, but, with a decrease in the SS filling rate, the pulling force of the SS on the soluble sugar in the vascular bundles decreases, and the sucrose flowing through the SS rachillae also decreases. The soluble sugar content in the IS rachillae also changes similarly. In summary, it is apparent that the development process of SEs in rachillae is closely related to the content of soluble sugar in rachillae and the dynamics of grain filling. This provides additional evidence that the underdeveloped vascular bundles of IS rachillae may be one of the reasons for the poor seed setting rate and low plumpness of the IS.

## Conclusion

The large-panicle japonica rice strains W1844 and WJ165 have large differences in grain weight and plumpness between the SS and IS. In this study, the vascular bundle structures of the branches in different positions of the panicle were observed, and their cross-sectional areas were calculated using an OM and an SEM. The results showed that vascular bundle cross-sectional areas of the basal primary branches were greater than those in the upper primary branches; moreover, there was only a slight difference between the basal secondary branches and the upper secondary branches in the cross-sectional areas of vascular bundles. However, the vascular bundle cross-sectional areas of the IS rachillae were lower than those in the SS rachillae. Therefore, we believe that the underdeveloped vascular bundles of IS rachillae may be the limiting factor of IS filling. In addition, during the process of rice grain filling, the ultrastructure of SEs, CCs, and PCs in the central large vascular bundle of the SS rachillae and IS rachillae was observed, and the soluble sugar and protein contents of the SS rachillae and IS rachillae were determined. The results showed that the developmental process of the SEs and the CCs in the central large vascular bundle of the SS rachillae and IS rachillae is similar. However, in the early stage of SS filling, there are a large number of M and PD in the CCs, which then degenerate and disappear. Therefore, it is believed that the physiological function of the rachillae vascular bundle mainly contributes to the grain filling at the initial filling stage.

## Data Availability Statement

The original contributions presented in the study are included in the article/supplementary material, further inquiries can be directed to the corresponding author/s.

## Author Contributions

CY, LW, and HH designed the experiments. CY, HW, YH, PX, JG, and FY performed part of the experiments. CY, LZ, and XZ analyzed experimental results. CY, HH, and JK prepared the manuscript. All the authors have read and approved the final manuscript.

## Conflict of Interest

The authors declare that the research was conducted in the absence of any commercial or financial relationships that could be construed as a potential conflict of interest.

## Publisher’s Note

All claims expressed in this article are solely those of the authors and do not necessarily represent those of their affiliated organizations, or those of the publisher, the editors and the reviewers. Any product that may be evaluated in this article, or claim that may be made by its manufacturer, is not guaranteed or endorsed by the publisher.

## Abbreviations

CC: companion cell
CH: chloroplast
CW: cell wall
DPA: days post anthesis
ChlC: chlorenchyma cell
IS: inferior spikelets
LVB: large vascular bundle
M: mitochondria
N: nucleus
OM: optical microscopy
P: phloem
PBS: phosphate buffered saline
PC: parenchyma cell
PD: plasmodesmata
PT: parenchymatous tissue
SC: sclerenchymatous cell
SE: sieve element
SEM: scanning electron microscopy
SS: superior spikelets
SVB: small vascular bundle
T: tube
TEM: transmission electron microscopy
V: vacuole
VBS: vascular bundle sheath
X: xylem.
